# On the relationship among different motor processes: a computational modeling approach

**DOI:** 10.3389/fncom.2015.00034

**Published:** 2015-03-18

**Authors:** Ahmed A. Moustafa

**Affiliations:** Department of Veterans Affairs, Marcs Institute for Brain and Behaviour and School of Social Sciences and Psychology, University of Western SydneySydney, NSW, Australia

**Keywords:** motor effectors, Parkinson's disease, dopamine, computational modeling, sequence learning

In a recent study in *Neurological Science*, Park and colleagues investigated the relationship among gait and speech disorders in patients with Parkinson's disease (PD) (Park et al., 2014). They found that gait disturbance in PD patients correlate with speech problems. Specially, they found that gait velocity correlates with initiation of words during speech and stride length correlates with speech rate in the patients. As reported in the Park and colleagues study (Park et al., 2014), other studies correlated gait with speech in PD patients (Giladi et al., 2001; Goberman, 2005; Moreau et al., 2007; Cantiniaux et al., 2010; Nutt et al., 2011). As similar to Park and colleagues' study, speech disturbance and slow hand movements were also found to correlate in PD patients (Skodda et al., 2011), but see Maillet et al. (2012) for different results on the relationship between hand movement control and speech production in PD. Unlike prior studies, Park and colleagues also found that cueing (the use of visual and auditory cues to enhance motor output) has similar effects on changing speech and gait parameters in PD patients. This findings stress the importance of perceptual processing on motor production, and the possibility of treating motor deficits in PD patients by augmenting perceptual processes.

The findings on the relationship among different motor processes were previously reported in the literature in healthy subjects as well as patient populations. For example, one study found an overlapped representation in the brain for hand and leg movement (Ehrsson et al., 2000). Lewis and colleagues found that freezing of gait (difficulty walking despite the attempt to move forward which is often described as being glued to the ground) correlates with freezing of hand movements in patients with PD (Naismith and Lewis, 2010). Nieuwboer and colleagues have also found evidence that the control of foot and leg movements do correlate in PD patients (Vercruysse et al., 2012). Studies also found impaired handwriting correlates with the severity of motor symptoms in PD (Wagle Shukla et al., 2012). Similarly, it was found that speech production in PD patients correlates with other motor processes including gait, facial movements, and postural control (Goberman, 2005). Studies have also reported a correlation between saccadic eye movements and finger and body movement in PD patients (Shibasaki et al., 1979). Hand control impairment were also found to correlate with other motor processes in disorders such as dystonia (Nowak and Hermsdörfer, 2005) and Huntington Disease Gordon et al. (2000).

Nonetheless, research on the relationship among different motor effectors is rather limited. Most researchers focus on only one kind of motor process—gait, speech, handwriting, eye movement or other. Thus, there is a dearth of knowledge on how the brain controls the different motor effectors, and whether they are similar or not. Further, it is not clear how and why such processes correlate. Park and colleagues suggest that executive and perceptual dysfunction may underlie both gait and speech deficits in PD, and that sensory cortex projections to the basal ganglia may be the neural mechanism underlying these deficits (Park et al., 2014).

Computational modeling studies have the potential to explain why such motor processes correlate. Computational modeling approaches suggest that both gait, reaching, handwriting, and speech share more or less the same elemental motor processes, which include the selection of appropriate motor actions at every time step, sequencing of movements, coordination of different motor effectors, as well as correct performance of these responses. Other relevant processes include suppression of alternative motor plans (Aron et al., 2007), a process that plays a key role in successful motor production. Some of these processes were found to rely on separable neural structures, including the basal ganglia, premotor cortex, motor cortex, prefrontal cortex, and the cerebellum (Bullock et al., 2009; Bordner et al., 2011; Gershman et al., 2014; Husarova et al., 2014; Kishore et al., 2014; Schulz et al., 2014). While the basal ganglia is assumed to play a key role in action selection (Gurney et al., 2001), the cerebellum is hypothesized to play a role in motor coordination (Kashiwabuchi et al., 1995; Shibuki et al., 1996) and timing (Ivry et al., 2002; Spencer and Ivry, 2005; Spencer et al., 2005; Schlerf et al., 2007).

Motor cortical areas were repeatedly found to play a role in sequencing and maintaining motor plans in working memory to actively execute a plan (Dagher et al., 1999). Future computational models should address how interactions among these brain structures explain performance in different motor processes (gait, handwriting, reaching, speech, among others). One such modeling framework that can be used to simulate complex motor processes is one proposed by Houk et al. (2007). Although this model was not applied to specific motor processes, it explains the information processing mechanism underlying the interactions of the basal ganglia, cerebellum, and cortex. Other class of computational models were shown to simulate various motor outputs (Gangadhar et al., 2009; Gupta et al., 2013; Muralidharan et al., 2014), although these models did not explain how these motor processes relate to each other, and were mostly focused on basal ganglia function. Computational modeling approaches have the potential to explain the similarities and differences among the different motor effectors, as found in Park et al. (2014). Importantly, one potential explanation for the similarities and differences among the different motor processes could be explained by the motor hierarchy hypothesis (Botvinick, 2008), which argues that different motor cortical and prefrontal areas are involved in hierarchical motor control. One potential modeling framework to simulate hierarchical model is provided by Stringer and Rolls (2007), which shows how we can learn and initiate sequence of motor responses. It is possible that motor execution occurs at view stages, starting from selection to response, and that the different motor processes rely on common early motor processes (e.g., action selection and sequencing), but possibly differ on the motor execution mechanism. Future neuroimaging and modeling work should confirm or disconfirm these relationships.

## Conflict of interest statement

The author declares that the research was conducted in the absence of any commercial or financial relationships that could be construed as a potential conflict of interest.
